# Supplementary material for: Immune Responses Induced by a Recombinant Lactiplantibacillus plantarum Surface-Displaying the gD Protein of Pseudorabies Virus
Source: Viruses. 2024 Jul 24;16(8):1189. doi: 10.3390/v16081189 (PMC11359135; doi:10.3390/v16081189)
Supplement: Supplementary file 1 [file viruses-16-01189-s001.zip › viruses-3035916-supplementary.docx]

**Supplementary information**

The amino acid sequence of LP3065 contains three distinct LPxTG peptide segments. These distinct parts are marked, and provided as below.

Signal peptide (underline), LPXTG motif (overstriking), and more than three positively charged amino acids (shadow), R or K.

MPNKWWRLILGVMLVLSWAIPVRAATHGRTEISVGFYETKYSADRIIPARVIPDGGHPYVIRPADPGTQGQRNQSERKPIRRQLIIDGWLRGQSRNGQQ**LPQTSE**RLVIGSILGGMGLLASVLAGALYRILKRQDGGERNA.
